# Benzalkonium chloride disinfectant residues stimulate biofilm formation and increase survival of *Vibrio* bacterial pathogens

**DOI:** 10.3389/fmicb.2023.1309032

**Published:** 2024-02-07

**Authors:** Julia Mougin, Graziella Midelet, Sophie Leterme, Giles Best, Timothy Ells, Alyssa Joyce, Harriet Whiley, Thomas Brauge

**Affiliations:** ^1^Department of Marine Sciences, University of Gothenburg, Gothenburg, Sweden; ^2^Bacteriology and Parasitology of Fishery and Aquaculture Products Unit, Laboratory for Food Safety, ANSES, Boulogne-sur-Mer, France; ^3^College of Science and Engineering, Flinders University, Adelaide, SA, Australia; ^4^ARC Training Centre for Biofilm Research and Innovation, Flinders University, Adelaide, SA, Australia; ^5^Flinders Institute for NanoScale Science and Technology, Flinders University, Adelaide, SA, Australia; ^6^Flinders Health and Medical Research Institute (FHMRI), College of Medicine and Public Health, Flinders University, Adelaide, SA, Australia; ^7^Agriculture and Agri-Food Canada, Kentville Research and Development Centre, Kentville, NS, Canada

**Keywords:** benzalkonium chloride, biocide, *Vibrio*, biofilm, viability, c-di-GMP, seafood, aquaculture

## Abstract

*Vibrio* spp. are opportunistic human and animal pathogens found ubiquitously in marine environments. Globally, there is a predicted rise in the prevalence of *Vibrio* spp. due to increasing ocean temperatures, which carries significant implications for public health and the seafood industry. Consequently, there is an urgent need for enhanced strategies to control *Vibrio* spp. and prevent contamination, particularly in aquaculture and seafood processing facilities. Presently, these industries employ various disinfectants, including benzalkonium chloride (BAC), as part of their management strategies. While higher concentrations of BAC may be effective against these pathogens, inadequate rinsing post-disinfection could result in residual concentrations of BAC in the surrounding environment. This study aimed to investigate the adaptation and survival of *Vibrio* spp. exposed to varying concentrations of BAC residues. Results revealed that *Vibrio* bacteria, when exposed, exhibited a phenotypic adaptation characterized by an increase in biofilm biomass. Importantly, this effect was found to be strain-specific rather than species-specific. Exposure to BAC residues induced physiological changes in *Vibrio* biofilms, leading to an increase in the number of injured and alive cells within the biofilm. The exact nature of the “injured” bacteria remains unclear, but it is postulated that BAC might heighten the risk of viable but non-culturable (VBNC) bacteria development. These VBNC bacteria pose a significant threat, especially since they cannot be detected using the standard culture-based methods commonly employed for microbiological risk assessment in aquaculture and seafood industries. The undetected presence of VBNC bacteria could result in recurrent contamination events and subsequent disease outbreaks. This study provides evidence regarding the role of c-di-GMP signaling pathways in *Vibrio* adaptation mechanisms and suggests that c-di-GMP mediated repression is a potential avenue for further research. The findings underscore that the misuse and overuse of BAC may increase the risk of biofilm development and bacterial survival within the seafood processing chain.

## 1 Introduction

In the seafood industry, *Vibrio* spp. are opportunistic bacterial pathogens and the causative agent of vibriosis in both animals and humans (Ashrafudoulla et al., 2021; Brauge et al., 2024). They are responsible for substantial economic losses, as they are a significant yield-limiting factor, as well as, a potential zoonotic and foodborne pathogen (Iwamoto et al., 2010). *Vibrio* spp. are ubiquitous in aquatic environments, resulting in their high prevalence in seafood products and the subsequent contamination of aquaculture and processing facilities. Survival of pathogenic *Vibrio* spp. in such environments, predominantly in biofilm form, has been identified as a source of cross-contamination, re-contamination, and recurring outbreaks (Mougin et al., 2019; Arunkumar et al., 2020; Mougin et al., 2021). Biofilms are complex structured microbial communities, either mono-or multispecies, that are attached to a surface and embedded within a self-produced protective matrix of Extracellular Polymeric Substances (EPSs) (Flemming and Wingender, 2010). They are the predominant form of prokaryote life, with the switch from a planktonic to a robust sessile biofilm lifestyle entailing a cascade of pivotal proteins with roles in flagellar and pili biosynthesis, or polysaccharide production (Yildiz and Visick, 2009), regulated by complex mechanisms involving bis-(3′-5′)-cyclic dimeric guanosine monophosphate (c-di-GMP) signaling pathways, quorum sensing systems, and sigma factors (Yildiz and Visick, 2009; Homma and Kojima, 2022). Such formations jeopardize microbiological safety by providing microbial pathogens an ideal protection against disinfectants and antibiotics, which could lead to therapeutic failure (Bridier et al., 2015; Carrascosa et al., 2021).

One of the challenges with assessing the effectiveness of strategies to manage *Vibrio* spp. within the seafood industry is its ability to enter the viable but non culturable (VBNC) state that cannot be detected by standard culture-based methods (Griffitt et al., 2011; Brauge et al., 2020). Previous research has shown environmental stressors, such as low nutrients and low temperatures can induce *Vibrio* spp. to transform into the VBNC state (Kaneko and Colwell, 1974; Oliver et al., 1995; Ramaiah et al., 2002). Cells in the VBNC state have been described as dormant or injured and have reduced metabolic activity; however, they have also been shown to retain their virulence and have an increased ability to survive unfavorable environmental conditions through metabolic and physiological changes (Griffitt et al., 2011). Furthermore, this state is reversible, meaning that under more favorable conditions, bacteria can transform back to the culturable state. This has implications for the management and control of *Vibrio* in the environment as VBNC *Vibrio* spp. will go undetected by the culture based method traditionally utilized by biosecurity and food safety management practices and the associated regulatory requirements and they also have increased ability to persist under unfavorable conditions (Bonnin-Jusserand et al., 2017). As such, the evaluation of improved biosecurity approaches to control *Vibrio* spp. in the environment and food processing facilities must consider all forms of *Vibrio* and not just the culturable state (Ayrapetyan and Oliver, 2016; Gao et al., 2021).

In this context, biosecurity management necessitates a regular and thorough decontamination of surfaces, including aquaculture tanks and food processing facilities, to avoid biofilm development and pathogen persistence. Disinfectants provide a broad spectrum of antimicrobial activity against bacteria, fungi and viruses, with benzalkonium chloride (BAC) being widely used across industries (Pereira and Tagkopoulos, 2019). BAC is a cationic surfactant consisting of quaternary ammonium compounds (QACs), with antimicrobial activity associated with disruption of bacterial membrane leading to leakage of intercellular elements (Ferreira et al., 2011; Dan et al., 2022). While an optimal BAC concentration might be effective to control pathogens, inadequate rinsing after disinfection, could lead to discharge of sublethal concentrations of BAC that will persist into the surrounding environment. Frequent exposure to non-toxic concentrations might induce a selection pressure over bacteria to evolve toward a decreased susceptibility to biocides (Oniciuc et al., 2019). The mechanism behind this phenomenon is still not fully understood, nevertheless several factors including change in membrane composition, upregulation of efflux pumps, downregulation of porins, biofilm formation or horizontal gene transfer (HGT) have proven to be involved in the resistance/tolerance process (Pereira and Tagkopoulos, 2019).

While the effect of sublethal BAC concentrations on various human pathogens, including for instance *Escherichia coli*, *Klebsiella pneumoniae*, *Listeria monocytogenes*, *Pseudomonas aeruginosa*, or *Staphylococcus epidermidis*, has been intensively researched (Houari and Di Martino, 2007; Pagedar et al., 2012; Brauge et al., 2020), no study has investigated the effect on *Vibrio* spp. Given that BAC is one of the most commonly used disinfectants in aquaculture and seafood processing facilities, understanding the adaptation and tolerance mechanisms of *Vibrio* spp. against BAC, is of paramount importance. This information on *Vibrio* spp. evolution and adaptation mechanisms will inform the future development of effective biocontrol strategies.

This study addressed this gap in knowledge and investigated the impact of BAC residues on both biofilm formation and pre-existing biofilms across several *Vibrio* species that are of relevance to the seafood industry. Biofilm biomass was quantified using Crystal Violet (CV) staining, alongside cellular metabolic activity using 2,3,5-triphenyl tetrazolium chloride (TTC) colorimetric assay. Survival of alive and injured *Vibrio* exposed to BAC residues was investigated through flow cytometry and fluorescence microscopy, using viability dyes. To delve into the underlying mechanism, modulation of c-di-GMP signaling pathways under BAC residues was monitored.

## 2 Materials and methods

### 2.1 Bacterial isolates and culture conditions

A total of 6 *Vibrio* isolates, widely recognized as human and/or animal pathogens within the *Vibrio* genus, were used in this study (Table 1). Isolates were obtained from the Veterinary Diagnostic Laboratory (VDL) aquatic collection conserved at the University of Adelaide (South-Australia). These bacteria had previously been isolated from various aquatic organisms and identified by matrix-assisted laser desorption ionization/time-of-flight mass spectrometry (MALDI TOF MS). Before all experiments, bacteria were sub-cultured on Marine Agar (MA; US Biological Life Sciences, Salem, MA, USA) and incubated overnight at 22°C. For *Vibrio cholerae*-WV30 isolates, plates were incubated for 48 h to ensure growth of visible colonies. Following the prescribed incubation period for each culture, a single colony forming unit (CFU) was selected and transferred into 25 mL Marine Broth (MB; Difco, Detroit, MI, USA), which was in turn, incubated overnight at 22°C.

**Table 1:** List of bacterial isolates used in this study.

Isolate ID	Assigned species	Host/origin	Isolation place	Isolation date
Vp-QV7	*Vibrio parahaemolyticus*	Crustacean/Haemolymph	Queensland, Australia	2000
Vp-QV9	*Vibrio parahaemolyticus*	Crustacean/Haemolymph	Queensland, Australia	2003
Va-WV43	*Vibrio alginolyticus*	Seahorse/Skin	-	2004
Va-DV20	*Vibrio alginolyticus*	Mud crab larval rearing tank/Water	Darwin, Australia	2002
Vh-WV45	*Vibrio harveyi*	Seahorse/Skin	-	2004
Vc-WV30	*Vibrio cholerae*	Lung fish/Flank	Perth, Australia	2004

### 2.2 Biofilm inhibition treatment

The effect of disinfectant residues on biofilm formation was assessed under static conditions following a previously described protocol (Haney et al., 2018, 2021) with some modifications. The experiments were conducted in 96-well flat bottom polystyrene microtitre plates (353,072 – BD Falcon, Durham, NC, USA). A volume of 90 μL of *Vibrio* bacterial suspension at 10^8^ CFU.mL^−1^ or sterile MB media was added to the wells. Then, 10 μL of either sterile distilled water or benzalkonium chloride (BAC) disinfectant solutions prepared in sterile distilled water (stock solution at 1.6% w/v or 16 g.L^−1^) were added, resulting in final concentrations of 1.25, 0.63, 0.31, 0.16, 0.08, 0.04, 0.02, and 0.01% v/v, corresponding to 200 mg.L^−1^, 100 mg.L^−1^, 50 mg.L^−1^, 25 mg.L^−1^, 12.5 mg.L^−1^, 6.25 mg.L^−1^, 3.13 mg.L^−1^. Sterile MB media containing 10 μL BAC disinfectant was used as a negative control, and bacterial suspension at 10^8^ CFU.mL^−1^ in 10 μL of sterile distilled water was used as a positive control. After incubation for 24 h at 22°C, planktonic bacteria were removed and the remaining bacterial biomass was washed three times with 200 μL sterile distilled water. Each plate contained two replicate wells for each bacterial suspension and disinfectant treatment combination, with peripheral wells being filled with 200 μL sterile distilled water to reduce water loss due to evaporation. Each plate setup was performed in duplicate, thus for each treatment condition a total of four replicates were obtained.

### 2.3 Biofilm eradication treatment

To evaluate the effect of disinfectant residuals on established biofilms, 24 h biofilms were prepared as previously described in section 3.2 without the presence of BAC, then treated with the different concentrations of disinfectant, ranging from 1.25 to 0.01% BAC. After 24 h of incubation at 22°C, planktonic bacteria were removed from the wells and the adherent bacterial biomass was washed three times with 200 μL sterile distilled water. Then, 180 μL of sterile MB media was added in each well, followed by 20 μL of disinfectant solutions at different concentrations or distilled water. Similar to the experiment described in section 3.2, sterile MB media containing 20 μL of disinfectant was added to wells with no pre-formed biofilm, which served as the negative control, and the treatment of pre-formed biofilm with sterile MB media containing 20 μL of distilled water was used as the positive control. After incubation for another 24 h at 22°C, planktonic bacteria were removed and the wells were washed three times with 200 μL sterile distilled water.

### 2.4 Biofilm analysis

#### 2.4.1 CV staining

CV staining was performed as described by Haney et al. (2021), with slight modifications. Adhered biomass was stained with 200 μL of 0.1% Crystal Violet (CV; Sigma-Aldrich, St. Louis, MO, USA) solution for 20 min, and then washed three times with 200 μL of sterile distilled water. After air drying for 5 min, 200 μL of 70% ethanol solution was added to each well and the bound CV dye was eluted by gently pipetting up and down. The absorbance at 595 nm (A_585_) was measured using a SPECTROstar Nano microplate reader (BMG Labtech, Offenburg, Germany). Results are expressed as the mean of at least four biological replicates.

#### 2.4.2 TTC colorimetry

TTC colorimetric assay was performed as described by Haney et al. (2021), with minor modifications. Metabolic activity of the adhered cells was evaluated using a solution of 5% 2,3,5-triphenyl tetrazolium chloride stock solution (TTC; Sigma-Aldrich), prepared in sterile distilled water and sterilized by filtration through a 0.2 μm filter.

For the biofilm inhibition assay, 1 μL of 5% TTC stock was added to the MB medium for a final volume of 100 μL (i.e., final concentration 0.05%) in each well, then the plates were incubated for 24 h at 22°C. After BAC treatment and washing steps, as described in 3.2, TTC dye in the adhered biomass was dissolved by adding 200 μL of methanol to each well with gentle mixing. Quantitation of reduced TTC was assessed by reading the absorbance at 500 nm (A_500_) using a SPECTROstar Nano microplate reader (BMG Labtech).

For the biofilm eradication assay, pre-formed biofilms were grown for 24 h at 22°C, then washed as described in 3.3. Metabolic activity was evaluated, by adding 2 μL of stock TTC to each well containing 198 μL of MB medium (TTC final concentration 0.05%). After BAC treatment and washing steps, the TTC assay was carried out as described in the biofilm inhibition experiments above. Results are expressed as the mean of at least four biological replicates.

#### 2.4.3 Epifluorescence microscopy

After biofilm inhibition or eradication treatments described in sections 3.3 and 3.4, cell viability was assessed using a LIVE/DEAD® BacLight™ bacterial viability kit (Invitrogen, Carlsbad, CA, USA), where 20 μL of the working solution containing SYTO™ 9 and propidium iodide dyes was deposited into each well and then incubated in the dark for 15 min at room temperature. Visualization of the stained biofilm was performed using an epifluorescence microscope (Imager.Z1, Zeiss, Marly-le-Roi, France) connected to a CCD camera (Axiocam – MRm, Zeiss) with ×40 and ×100 magnification. A minimum of four random acquisitions were recorded for each well.

#### 2.4.4 Flow cytometry

Quantification of viable, dead and damaged bacteria was performed by flow cytometry according to the protocol of Nisar et al. (2023), using the BD™ Cell Viability kit and liquid counting beads (349,480, Becton Dickinson and BD Biosciences, San Jose, CA, USA), with slight modifications.

Briefly, after biofilm formation and inhibition or eradication treatments as described above (3.3 and 3.4), adhered bacteria were removed from the wells using a sterile cotton swab (Deltalab, BCN, Spain). The swab was then placed into 1 mL sterile physiological saline, and vortexed for 1 min to resuspend the cells. To confirm biofilm removal, the wells were stained with CV after swabbing and observed using a light microscope.

A volume of 200 μL of filter sterilized staining buffer (1 mM EDTA and 0.01% tween-20 in 1X PBS, pH 7.4 ± 0.1) was then added to 200 μL of cell suspension. Cell staining was performed by adding 4 μL of thiazole orange (TO; 420 nM final concentration) and 4 μL of propidium iodide (PI; 48 μM final concentration). After incubation for 15 min, 50 μL of counting bead suspension was added to the solution.

Analysis was performed on a BD FACSAria™ Fusion flow cytometer (BD Biosciences). For each sample, cell parameters were determined on at least 20,000 events, and cell viability (i.e., alive, dead and injured cells) was determined according to their fluorescence properties. Debris were excluded based on forward (size) and side (internal complexity) scatter properties. Each assay was performed with several controls to accurately gate the different populations. As a control for dead and injured cells, a cell suspension was prepared from a 10^8^ CFU mL^−1^ *Vibrio* bacterial suspension that had been heat-treated for 30 min at 75°C; a control containing predominantly live cells, was prepared from a 24 h *Vibrio* culture that was adjusted to 10^8^ CFU mL^−1^; and stained/unstained blank controls without any cells were prepared in sterile physiological saline. Results are expressed as the mean of at least three biological replicates.

#### 2.4.5 c-di-GMP measurement

The treated and washed biofilms were lysed using 200 μL of Bacterial Protein Extraction Reagent (B-PER) (Thermo Fisher Scientific, Waltham, MA, USA), and incubated at room temperature for 10 min. After centrifugation at 5,000 × *g* for 10 min, the resulting supernatant was used to measure the intracellular concentration of c-di-GMP using a specific c-di-GMP ELISA kit (Cayman Chemical, Ann Arbor, Michigan, USA), following the manufacturer’s instructions. Intracellular c-di-GMP concentrations were determined using a standard curve generated with a standard provided in the kit. Results are expressed as the mean of at least three biological replicates.

### 2.5 Statistical analysis

Differences in relative cell proportions (i.e., dead, injured, alive) and bacterial c-di-GMP production between BAC treatments were evaluated using a Kruskal-Wallis test followed by a *post hoc* Dunn test with Bonferroni correction, since normality and homoscedasticity of the distribution hypothesis were not verified. The differences were considered statistically significant at value of *p* < 0.05 (*n* = 3). Statistical analyses were performed using RStudio ver. 4.2.0 software (R Core Team, 2013). Graphics were drawn using the ggplot2 packages ver. 3.3.6 (Wickham, 2016).

## 3 Results

**Figure 1:**
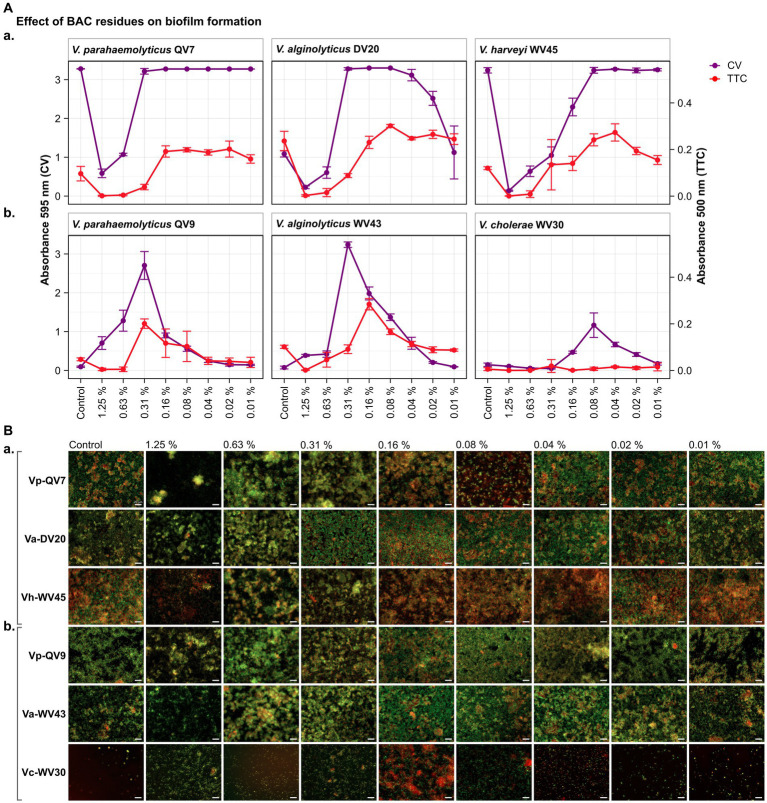
Impact of different benzalkonium chloride (BAC) concentrations on *Vibrio* biofilm formation in 96-well polystyrene microtiter plates after 24 h. BAC concentrations ranged from 0% (Control) to 1.25, 0.63, 0.31, 0.16, 0.08, 0.04, and 0.02%. A total of six *Vibrio* isolates were studied: including two strains of *Vibrio parahaemolyticus* (QV7 and QV9), two strains of *Vibrio alginolyticus* (DV20 and WV43), one strain of *Vibrio harveyi* (WV45) and one strain of *Vibrio cholerae* (WV30). **(A)** Comparisons of the effect of BAC on adhered bacterial biomass and its metabolic activity. Biofilm quantified by CV staining is indicated in purple (left axis) and metabolic activity quantified by TTC metabolism is indicated in red (right axis). Data points represent the mean of the absorbance at 595 nm (CV) or 500 nm (TTC) with associated error bars representing the standard deviation (*n* = 3). **(B)** Bacterial viability assessment by epifluorescence microscopy after BAC treatment. Biofilms were stained with the LIVE/DEAD® BacLight™ bacterial viability kit. Live cells are stained green with the primary SYTO 9 dye, while dead or injured cells with compromised membranes are stain red due to the secondary propidium iodide (PI) stain. The white scale bar represents 20 μm. Isolates were classified as panels **(Aa,Ba)** strong biofilm producers (Vp-QV7, Va-DV20, Vh-WV45) and panels **(Ab,Bb)** moderate biofilm producers (Vp-QV9, Va-WV43, Vc-WV30).

**Figure 2:**
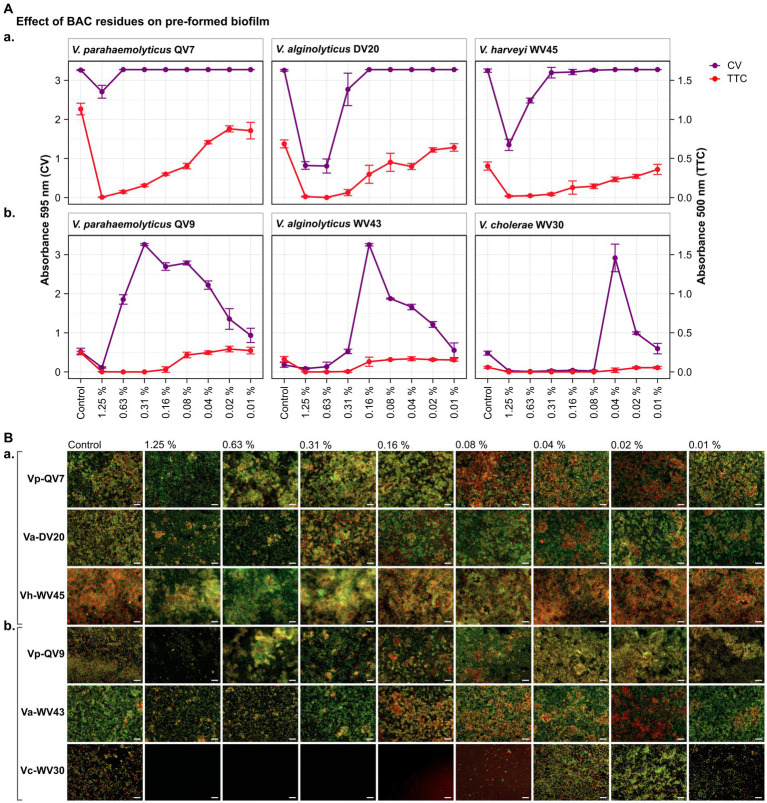
Impact of different benzalkonium chloride (BAC) concentrations on 24 h-preformed *Vibrio* biofilms cultured on 96-well polystyrene microtiter plates for 24 h. BAC concentrations ranged from 0% (Control) to 1.25, 0.63, 0.31, 0.16, 0.08, 0.04, and 0.02%. A total of six *Vibrio* isolates were studied: including two strains of *V. parahaemolyticus* (QV7 and QV9), two strains of *V. alginolyticus* (DV20 and WV43), one strain of *V. harveyi* (WV45) and one strain of *V. cholerae* (WV30). **(A)** Comparisons of the effect of BAC on adhered bacterial biomass and metabolic activity. Biofilm adhered biomass quantified by CV staining is indicated in purple (left axis) and metabolic activity quantified by TTC metabolism is indicated in red (right axis). Data points represent the mean of the absorbance at 595 nm (CV) or 500 nm (TTC) with associated error bars representing the standard deviation (*n* = 3). **(B)** Bacterial viability assessment by epifluorescence microscopy after disinfectant treatment. Biofilms were stained with the LIVE/DEAD® BacLight™ bacterial viability kit. Live cells are stained green with the primary SYTO 9 dye while dead or injured cells with compromised membranes are stained red due to the secondary propidium iodide (PI) stain. The white scale bar represents 20 μm. Isolates were classified as panels **(Aa,Ba)** strong biofilm producers (Vp-QV7, Va-DV20, Vh-WV45) and panels **(Ab,Bb)** moderate biofilm producers (Vp-QV9, Va-WV43, Vc-WV30).

As highlighted by the CV staining assay (control treatments shown in Figures 1A, 2A), bacterial isolates displayed a distinct growth capacity in the form of a biofilm after 24 h of incubation. The highest levels of adhered biomass were observed for *Vibrio parahaemolyticus* QV7, *Vibrio alginolyticus* DV20 and *Vibrio harveyi* WV45, hereafter referred to as strong biofilm producers, with mean CV absorbance values ranging from 3.28 to 1.09 (mean 2.53 ± 1.25), (control treatments shown in Figures 1, 2Aa). Conversely, low adhered biomass was observed for *Vibrio parahaemolyticus* QV9, *Vibrio alginolyticus* WV43 and *Vibrio cholerae* WV30, hereafter referred to as moderate biofilm producers, with mean values ranging from 0.15 to 0.07 (mean 0.11 ± 0.04), (control treatments shown Figures 1, 2Ab).

### 3.1 Biofilm formation in presence of BAC residues

#### 3.1.1 CV staining

As expected, for strong biofilm producers, in particular Vp-QV7 and Vh-WV45, BAC was able to disrupt biofilm formation in a concentration-dependent manner, as shown by the CV staining assay (Figure 1Aa). The highest concentration of BAC 1.25% led to 82.1 ± 3.4%, 79.1 ± 3.3%, and 95.7 ± 0.5% reductions in biofilm relative to the control, for Vp-QV7, Va-DV20, and Vh-WV45, respectively. In contrast, at concentrations of BAC ≤ 0.31%, no significant effect on biofilm formation was observed for strains Vp-QV7 or Vh-WV45 biofilm formation. However, for the Va-DV20 strain which had the thinnest biofilms among the “strong” producers group, concentrations ranging from 0.31 to 0.02% stimulated biofilm production as biomass increases were between 3-and 2.3-fold, compared with controls.

Surprisingly, for the moderate biofilm producers, BAC stimulated biofilm formation following a parabolic relationship between increasing biofilm density and the concentration of disinfectant (Figure 1Ab). The highest amount of adhered biomass was detected at a BAC concentration of 0.31% for both Vp-QV9 and Va-WV43, while for Vc-WV30 the BAC concentration was 0.08%. As such, the adhered biomass increased by a factor of 28.6 for Vp-QV9, and 43.8 for Va-WV43, as well as 7.9 for Vc-WV30. Interestingly, at the highest BAC concentrations (i.e., 1.25 and 0.63%) the two strongest biofilm-forming strains (Vp-QV7 and Vh-WV45) displayed the greatest reductions in adhered biomass, relative to their respective controls; but at 0.31% BAC, maximum biofilm formation was restored. In contrast, for moderate-producers Vp-QV9 and Va-WV43, these high BAC levels promoted biofilm production, which peaked at 0.31% BAC, the same concentration at which full biofilm production was restored in the aforementioned strong-producer strains.

#### 3.1.2 TTC colorimetric assay

According to the TTC colorimetry assay, for strong biofilm producers (Figure 1Aa), as the concentration of BAC increased, the metabolic activity decreased for all tested isolates, as expected. The highest concentration of BAC (1.25%), led to 98.7 ± 2.3%, 99.0 ± 1.5% and 99.7 ± 0.7% reductions in metabolic activity for Vp-QV7, Va-DV20 and Vh-WV45, respectively compared with their controls. However, comparisons of CV staining and TTC metabolism quantification demonstrated that high absorbance readings for adhered biomass do not necessarily coincide with high readings for metabolic activity, as observed at 0.31% BAC for both Vp-QV7 and Va-DV20.

For moderate biofilm producers (Figure 1Ab), results for the TTC colorimetry assay showed a parabolic behavior for strains Vp-QV9 and Va-WV43 when exposed to the gradient of BAC concentrations, while no measurable metabolic activity was detected for Vc-WV30, even at 0.08% BAC, the concentration at which some biofilm formation was detected for this strain when stained with CV. Metabolic activity was enhanced by a factor of 4.2 for Vp-QV9 and 2.8 for Va-WV43 with 0.31% BAC, compared with their controls. However, similar to the observations for the strong biofilm strains, it was once again demonstrated that increased detection of adhered biomass does not necessarily correlate with increasing metabolic activity, as observed between 1.25 and 0.63% BAC for Vp-QV9, at 0.31% BAC for Va-WV43, as well as between 0.16 and 0.02% BAC for Vc-WV30.

#### 3.1.3 Microscopy

Results obtained from the CV and TTC assays were compared to epifluorescence microscopic observations after live/dead staining for viability assessment (Figure 1B). Overall, under all tested conditions, the observed biofilms were heterogeneous in structure displaying a mixture of both living bacteria (stained in green) and dead bacteria (stained in red). For strong biofilm producing strains Vp-QV7 and Vh-WV45 (Figure 1Ba), exposure to the highest concentrations of BAC at 1.25 and 0.63% led to the formation of aggregates consisting of both viable and dead cells, accompanied by a zone of free cells surrounding these aggregates. For these two strains, the cell density was reduced for treatments at 1.25%. For Va-DV20, the BAC concentrations that stimulated biofilm biomass production (i.e., 0.31 to 0.02%) led to more compact biofilm structures, compared with the controls. Similar structures were observed for moderate biofilm producers Vp-QV9 (Figure 1Ab), between 0.63 and 0.08% BAC, and Va-WV43, between 0.63 and 0.04% BAC. BAC residues that stimulate biomass production resulted in an increase in microcolonies for the treated biofilms, particularly at 0.16, and 0.08% BAC.

### 3.2 Effect of BAC residues on established biofilms

#### 3.2.1 CV staining

As expected, the CV staining assay demonstrated that BAC effectively decreased pre-formed biofilms among strong biofilm producers in a concentration-dependent manner (Figure 2Aa). Notably, the highest BAC concentration of 1.25% resulted in biofilm reductions of 17.0 ± 5.0%, 74.8 ± 3.0% and 58.5 ± 4.4% for Vp-QV7, Va-DV20 and Vh-WV45 respectively, compared with their controls. In contrast, concentrations of 0.63, 0.16 and 0.31% BAC had no effect on the biofilm formation of Vp-QV7, Va-DV20 and Vh-WV45, respectively.

Regarding the moderate biofilm producers (Figure 2Ab), BAC stimulated biomass production of pre-formed biofilm following a parabolic trend in relation to the level of the disinfectant. The highest amount of adhered biomass was noted at BAC concentrations of 0.31, 0.16, 0.04% for Vp-QV9, Va-WV43, and Vc-WV30, respectively. Adhered biofilm biomass was increased up to 6.2-and 15.8-fold for Vp-QV9 and Va-WV43 respectively, as well as 6.1-fold for Vc-WV30 in comparison to their controls.

#### 3.2.2 TTC staining

For the strong biofilm-producing strains, results obtained for the TTC colorimetry assay showed that as the concentration of BAC increased, the metabolic activity of biofilm cells decreased (Figure 2Aa). Specifically, the highest BAC concentration of 1.25%, led to 99.6 ± 0.2%, 98.2 ± 1.0% and 95.5 ± 1.7% reductions in metabolic activity for Vp-QV7, Va-DV20 and Vh-WV45, respectively, compared with their controls. However, similar to the results from experiments on the inhibition of biofilm development (i.e., section 4.1), comparisons of CV staining and TTC metabolism quantification for these biofilm eradication trials also demonstrated that high levels of adhered biomass do not always correspond with high metabolic activity, as observed at 0.63% BAC for Vp-QV7, or 0.31% BAC for Vh-WV45.

Likewise, for moderate biofilm producers (Figure 2Ab), increasing the BAC concentration resulted in a decrease in metabolic activity for strains Vp-QV9 and Va-WV43, while no notable difference in such activity was detected for Vc-WV30. The comparisons of results from CV staining and TTC metabolism quantification highlights again that an increased detection of adhered biomass does not necessarily correlate with an increase in metabolic activity, as observed at 0.31% BAC for Vp-QV9 or 0.04% BAC for Vc-WV30.

#### 3.2.3 Microscopy

As in section 4.1.3 for the inhibition studies, CV and TTC results obtained from the biofilm eradication experiments were also corroborated by epifluorescence microscopy using live/dead staining to assess bacterial cell viability (Figure 2B). Under all tested conditions, biofilms were again shown to consist of heterogenous mixture of a combination of live and dead bacteria, colored in green and red, respectively.

For strong biofilm-producing strains Vp-QV7, Va-DV20, and Vh-WV45 (Figures 2Ba,Bb), exposure to the highest BAC concentrations ≥0.31% led to the formation of aggregates consisting of both viable and dead cells, accompanied by a zone of free cells surrounding these aggregates. For these three strains, the biofilms treated at concentrations ≤0.16% resembling phenotypes similar to the control conditions confirmed the results from crystal violet and TTC assays.

In the case of moderate biofilm producers (Vp-QV9, Va-WV43, and Vc-WV30) (Figure 1Aa), higher BAC concentrations (1.25 and 0.63%) reduced the cell density within the biofilms for all three strains. The BAC concentrations that stimulated biofilm biomass production resulted in more compact structures compared with the controls. This was observed between 0.63 and 0.02% BAC for Vp-QV9, between 0.16 and 0.04% BAC for Va-WV43, and at 0.04 and 0.02% BAC for Vc-WV30.

### 3.3 Effect of BAC on the survival of *Vibrio*

**Figure 3:**
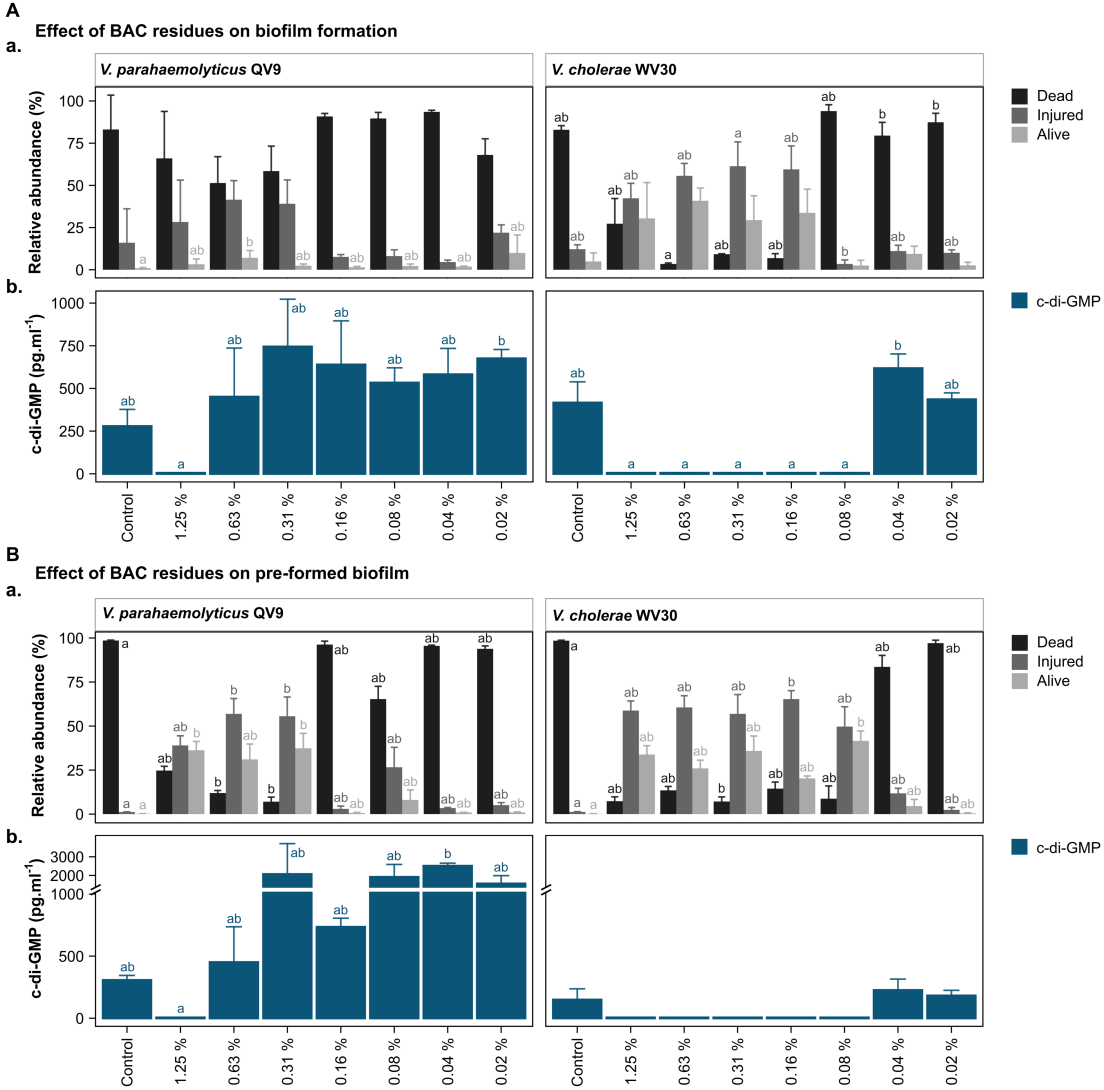
Bacterial viability assessment and c-di-GMP ELISA quantification after treatment of two *Vibrio* isolates with different concentrations of benzalkonium chloride (BAC). BAC concentrations ranged from 0% (Control) to 1.25, 0.63, 0.31, 0.16, 0.08, 0.04, and 0.02%. Two *Vibrio* isolates were selected for analysis: *V. parahaemolyticus* QV9 and *V. cholerae* WV30. The impact of BAC residues was evaluated for both **(A)**. biofilm formation and **(B)**. pre-formed biofilms. **(Aa,Ba)** Relative quantification of dead, injured and alive cells. Bacterial cells were classified as dead (black), injured (gray) and live intact (light gray) based on staining with thiazole orange and propidium iodide. Vertical bars represent the means of the relative abundance (%) ± standard deviation (*n* = 3). **(Ab,Bb)** Quantification of intracellular c-di-GMP after treatment with BAC. Vertical bars represent the mean of c-di-GMP concentration in pg.mL^−1^ ± standard deviation (*n* = 3), (detection limit <4.6 pg.mL^−1^). Different letters indicate significant differences between BAC treatments; Kruskal-Wallis, Dunn test; value of *p* < 0.05.

Two isolates, Vp-QV9 and Vc-WV30, which demonstrated the most notable biofilm overproduction when exposed to residual BAC, were chosen for further cell viability investigations using flow cytometry (Figure 3). Survival of bacteria after BAC treatment was evaluated on both biofilm formation (Figure 3Aa) and pre-formed biofilm (Figure 3Ba).

Overall, the 24 h biofilms for the untreated controls of both strains were comprised of more than 80% dead cells, while a further 16.0 ± 20.1% for Vp-QV9 and 12.1 ± 2.7% for Vc-WV30 were defined as injured. Only 0.9 ± 0.4% and 4.9 ± 5.1% of cells for Vp-QV9 and Vc-WV3, respectively, appeared to be alive and uninjured (Figure 3Aa). For Vp-QV9, the highest concentrations of BAC (i.e., 1.25, 0.63, and 0.31%) increased the proportion of injured cells within the biofilm, ranging from 28.3 ± 24.8 to 41.5 ± 11.3%. At 0.63% BAC, the relative quantity of alive cells was significantly higher, compared with the untreated control (Kruskal-Wallis, Dunn test; value of *p* = 0.0294). When Vc-WV30 was exposed to the same high BAC concentrations, the proportion of injured cells in the biofilm increased to a range of 42.3 ± 8.9% to 61.4 ± 14.4%, as did the range of alive cells (29.4 ± 14.5 to 40.8 ± 7.6%), whereas dead cells decreased to 27.2 ± 15.0 when exposed to 1.25% BAC and to less than 10% at BAC levels of 0.63, and 0.31%. For both isolates, the lowest BAC concentration had no effect on cell viability, relative to untreated controls.

The viability analysis on untreated pre-formed biofilms showed that for both Vp-QV9 and Vc-WV30, biofilm was composed of more than 98% dead cells, with 1.2 ± 0.1% and 0.2 ± 0.1% of the remaining cells identified as damaged or non-injured live cells, respectively (Figure 3Ba). For Vp-QV9, exposure to the highest BAC levels (i.e., 1.25, 0.63, and 0.31%) resulted in an increased proportion of injured cells within the biofilm ranging from 39.0 ± 5.5 to 57.0 ± 8.7%. At 0.63 and 0.31% BAC, the relative quantities of injured cells in the biofilms were significantly greater than those observed for the untreated controls (Kruskal-Wallis, Dunn test; value of *p* = 0.0290 and 0.0352, respectively), while the amount of dead cells was significantly reduced (value of *p* = 0.0394 and 0.0063 respectively). Similarly, at 1.25 and 0.31% BAC, the relative proportion of alive cells was significantly higher than that of the control (value of *p* = 0.0184). For Vc-WV30, BAC concentrations from 1.25 to 0.08% increase the proportion of injured cells, which ranged from 49.7 ± 11.3 to 65.3 ± 4.8%, as well as alive cells (20.3 ± 1.4 to 41.6 ± 5.7%), whereas dead cells decreased to approximately 15%. At 0.31% BAC, a significant reduction in dead cells within the biofilm was observed in comparison with those in the control biofilm (Kruskal-Wallis, Dunn test; value of *p* = 0.0444). Lowering the BAC level to 0.16% resulted in an increase in the proportion of injured cells (value of *p* = 0.0360) and decreasing the BAC concentration to 0.08% led to a significant reduction in the numbers of live intact cells (value of *p* = 0.0132). For both isolates, the lowest BAC concentration had no effect on cell viability, compared with the control.

### 3.4 c-di-GMP analysis

c-di-GMP levels in the biofilms were assessed for two isolates, Vp-QV9 and Vc-WV30 (Figure 3). c-di-GMP production was measured after biofilm formation in the presence of a range of BAC concentrations (Figure 3Ab), as well as in established biofilms following treatments with BAC at the same levels (Figure 3Bb).

For both biofilm formation and pre-formed biofilm assays, treatments of Vp-QV9 with 1.25% BAC resulted in a marked decrease in c-di-GMP levels compared with the control, whereas the lowest BAC concentrations led to an increase in c-di-GMP levels. However, these results were not statistically significant (value of *p* > 0.05). Similarly, for Vc-WV30, treatment with BAC concentrations ranging from 1.25 to 0.08% led to reduced c-di-GMP levels relative to those found in the control biofilms, while an increase was observed when exposed to lower BAC concentrations, albeit these changes were not statistically significant.

## 4 Discussion

The aim of this study was to investigate the effect of BAC residues at varying concentrations on the viability and development of biofilms of pathogenic *Vibrio* spp. which have importance in relation to the aquaculture and seafood industries. The study aimed to provide a deeper understanding of the potential risks for both human and animals associated with misuse/improper disinfectant usage or inadequate cleaning practices. Using six *Vibrio* isolates, it was demonstrated that BAC residues at certain concentrations could stimulate biofilm formation. Interestingly, this effect was strain-specific and was not consistently observed across all the strains tested. Subsequently, two of these *Vibrio* isolates were selected for a more in-depth analysis to help elucidate the mechanisms behind the survival adaptation and potential persistence of pathogenic *Vibrio* spp. along the seafood continuum. It was observed that there was an increase in the proportion of both injured and live intact cells within the biofilm in the presence of BAC residues. Furthermore, BAC treatments leading to increased biofilm biomass also exhibit c-di-GMP production, indicating a potential involvement of the c-di-GMP signaling pathway in bacterial adaptation. This study provides novel information pertaining to the potential impact of the misuse of BAC in aquaculture and in seafood processing industries, which could jeopardize animal and human health. Furthermore, this work provides new insights into the mechanisms involved in the adaptation and stress response of these bacteria when exposed to sub-lethal BAC residues, which is of paramount importance in order to develop innovative control strategies to improve food hygiene and overall safety of seafood products.

### 4.1 BAC residues induce *Vibrio* biofilm formation

To our knowledge, this study is the first to demonstrate that sub-lethal BAC residues may induce biofilm formation in *Vibrio* spp. We showed that after exposure to low BAC concentrations, *Vibrio* spp. harbor specific phenotypic responses depending on their inherent growth capacity associated with the biofilm state, and subsequent biofilm thickness. The greater the thickness of the biofilm, the less likely subinhibitory BAC concentrations will induce phenotypic changes, as characterized by an increase in biofilm biomass. Indeed, in a thick biofilm form, bacteria embedded in a self-protective matrix, are preserved from external factors, including disinfectants (Flemming and Wingender, 2010). As a result, the strongest biofilm producers tested in the current study displayed no biofilm induction since their thick biofilm offered ample protection against BAC. Bacteria with a planktonic lifestyle or those living within a thin biofilm, are more subject to inimical environmental changes including harmful interactions with chemical agents, and will thus need to adapt in order to survive. For example, for *P. aeruginosa* the protective role of biofilm against disinfectants was shown to be associated with the overexpression of biocide-degrading enzymes that were mediated by quorum sensing (Hassett et al., 1999). This could explain why, in our study weak biofilm producers switch from a thin to a thick biofilm, following exposure to sublethal concentrations of BAC. Similar to the results found in the current study, sub-lethal concentrations of BAC were able to induce biofilm formation by *Staphylococcus epidermidis* (Houari and Di Martino, 2007). In *Escherichia coli*, post-adaptation after disinfectant exposure also initiated significant changes in biofilm-forming ability (Pagedar et al., 2012). In agreement with our results, increased attachment of *P. aeruginosa* to polystyrene was observed following exposure to sub-lethal concentrations of sodium hypochlorite, which corresponded with a stimulation in c-di-GMP signaling (Strempel et al., 2017).

Several factors could explain the increased detection of biofilm biomass after BAC exposure, including elevated production of EPS, cell proliferation, and/or enhanced adhesion. Previously, it was demonstrated that unique changes in EPS composition and relative amounts occur in biofilms of *P. fluorescens* when exposed to subinhibitory concentrations of four different disinfectants (Dynes et al., 2009). Similarly, sublethal levels of chlorine dioxide induced an upregulation of genes involved in the production of EPS matrices in biofilms of *Bacillus subtilis* (Shemesh et al., 2010). The EPS matrix acts as a diffusion barrier for the biofilm, providing mechanical and/or chemical protection, that will prevent the transport of biocides to bacteria residing within. However, in a study investigating the effect of aminoglycoside antibiotics on biofilm formation, it was demonstrated that increased biofilm biomass after tobramycin-induction was due to an increase in CFU within the biofilm, rather than an increase in the EPS matrix (Hoffman et al., 2005).

Modification of the cell membrane and in particular increased surface hydrophobicity or changes in surface charge, were also demonstrated in Gram-negative bacteria after BAC adaptation (Braoudaki and Hilton, 2005). This phenomenon is related to protective processes to repel water-soluble compounds including disinfectants (Capita et al., 2014). Interestingly, hydrophobicity plays a key role in bacterial adhesion to hydrophobic surfaces. An increase in cell surface hydrophobicity could enhance bacterial adhesion and enhanced biofilm formation. Results from another study showed that the highest hydrophobicity values of *E. coli* were obtained after disinfectant adaptation, and these cells had the greatest potential to form biofilm (Capita et al., 2014).

In the current study, we measured increases in biofilm biomass through the implementation of a modified crystal violet (CV) staining assay. CV is an indicator of total attached biomass which not only binds to live, dead and VBNC cells within a biofilm, but it can also stain other components of the EPS matrix (Azeredo et al., 2017). It is therefore difficult to conclude whether the observed increase in absorbance levels associated with the biofilms is due to enhanced EPS production, cell multiplication, and/or greater cell adhesion. Therefore, such investigations require a combination of assays to gain a better understanding of the dynamics of biofilm formation. As such, we employed another colorimetric assay involving 2,3,5-triphenyl tetrazolium chloride (TTC), a substrate that yields a colored formazan derivative (red 1,3,5-triphenylformazan, TFP) after cellular reduction by dehydrogenases, in order to assess metabolic activity within the biofilm. Here, we demonstrated that an increase in biofilm biomass does not systematically correlate with increased metabolic activity. These results highlight that while changes in cell viability and/or multiplication might play a role in increasing biofilm biomass, the mechanisms are complex and involves different factors that may act concomitantly. Consequently, in order to better assess the effect of residual concentrations of BAC on cell viability within the biofilm, we further characterized and assessed the viability state.

### 4.2 BAC residues affect cell viability within *Vibrio* biofilm

Biofilms develop as complex heterogenous three-dimensional (3D) structures, comprising of different bacterial phenotypes. These dense formations induce a gradient of chemicals and nutrients, that contribute to physiological heterogeneity with the development of VBNC phenotypes (Bridier et al., 2011). VBNC bacteria, while still alive, are characterized by a reduced metabolic activity, and as a consequence, a reduction in susceptibility to environmental stressors such as disinfectants. Moreover, such resistant phenotypes are not able to divide and grow on traditional culture media, which is a major dilemma for the food industry since they cannot be detected using traditional standard culture-based biosecurity management procedures (Bonnin-Jusserand et al., 2017; Brauge et al., 2019). Consequently, apart from their resistance to disinfection processes, these bacteria will go undetected on the production chain, and may reach aquatic animals and human resulting in subsequent disease outbreaks.

Our study revealed that low concentrations of BAC can significantly affect bacterial viability, resulting in an increase in the relative proportion of both injured and intact live bacteria in the biofilms and a corresponding decrease in the number of dead cells. These determinations were achieved by two distinct methods based on live/dead staining to assess cellular viability; specifically, (i) epifluorescence microscopy for direct observation of *in vitro* biofilm cells, and (ii) and flow cytometry after biofilm detachment and dispersal. Both viability assays are based on membrane permeability and integrity, whereby the stains SYTO 9 and thiazole orange (TO) penetrate all bacterial membranes, while propidium iodide (PI) only penetrates bacteria with damaged membranes. As such, live cells will fluoresce green and dead bacteria will appear red. However, it has been reported that PI can significantly overestimate the number of dead bacteria when directly applied to biofilms since false layers of dead bacteria can be created due to the presence of extracellular DNA coating viable cells (Rosenberg et al., 2019). Therefore, the flow cytometry method offers an additional level of discrimination and detail, since biofilms must first be dislodged and aggregates of cells broken up. Moreover, an intermediate population, characterized as “injured” bacteria, can also be distinguished by this method due to distinct patterns of staining.

Previously, index sorting of heat shocked *Legionella pneumophila* resulted in a subpopulation of injured cells with reduced ATP contents, and loss of cultivability on standard growth media. In spite of this, these cells could be resuscitated in an amoeba host and proliferate, which may indicate the VBNC state (Nisar et al., 2023). Regarding the current study, further experiments are required to comprehensively characterize the perceived VBNC status of the *Vibrio* bacteria that were observed. However, reaching a definitive conclusion about the viability status of “injured” populations using viability staining combined with flow cytometry is challenging, as membrane permeability alone could not be used here as a reliable parameter to distinguish live and VBNC bacteria. Cells in this category exhibit signs of damage or stress, leading to alterations in membrane permeability, but their metabolic activity and ability for resuscitation should be further studied, to completely characterize those cells as VBNC bacteria.

### 4.3 c-di-GMP signaling pathway may regulate BAC-induced biofilms

As demonstrated here, the switch from a planktonic state to a robust sessile biofilm lifestyle under stress conditions is known to be a major phenotypic-specific response, not only leading to tolerance against biocides but, also to the persistence of foodborne pathogens within food processing facilities (Bridier et al., 2015). This process is known to be regulated by a complex system, involving different signaling pathways such as density-dependent quorum sensing or c-di-GMP signaling (Strempel et al., 2017). Our results demonstrated that increased biofilm biomass after subinhibitory BAC exposure was associated with high levels of intracellular bis-(3′-5′)-cyclic dimeric guanosine monophosphate (c-di-GMP), highlighting the major role of the c-di-GMP signaling network associated with disinfectant-tolerance mechanisms in *Vibrio* spp. c-di-GMP is an intracellular signaling molecule that regulates transition from a motile to a sessile biofilm lifestyle and *vice-versa*. It has been identified in a wide range of bacterial species including *Vibrio* (Tischler and Camilli, 2004), with modulation of diverse cellular functions such as cell motility, cell aggregation, adhesins biosynthesis, or production of exopolysaccharides. Such factors may induce an increase of biofilm biomass when c-di-GMP is high, while low levels will promote motility and biofilm dispersal (Römling et al., 2013).

Data here indicate that BAC residues may enhance the production of c-di-GMP, along with increased biofilm formation. Stress-related conditions due to the presence of disinfectant are known to modulate c-di-GMP levels, with bacteria benefiting by gaining a greater tolerance to biocides. For example, sublethal concentrations of hypochlorite (HClO) disinfectant have been shown to increase c-di-GMP levels in *P. aeruginosa*, with related increase of biofilm formation (Strempel et al., 2017). A c-di-GMP inducible exopolysaccharide in *Listeria monocytogenes* has also been shown to promote cell aggregation and tolerance against commonly used disinfectants (Chen et al., 2014). In addition, such signaling pathways might play a key role in pathogenesis and virulence mechanisms in a wide variety of bacteria (Valentini and Filloux, 2019). Of particular relevance, while the role of c-di-GMP and the mechanisms involved remain unclear, it has been demonstrated that this secondary messenger is associated with the regulation of some virulence genes in *V. cholerae* (Beyhan et al., 2006). Therefore, sublethal concentrations of disinfectant could not only lead to a potential persistence of pathogenic *Vibrio* spp. within seafood industries but could also result in a modification of virulence phenotype that may directly affect aquatic animals or humans and lead to disease outbreaks. In order to mitigate the risk potential associated with this phenomenon, a promising strategy could be realized through the use of c-di-GMP blockers that are aimed at inhibiting the c-di-GMP signaling pathway. Such molecules display great potential as antimicrobials with unexplored applications in the seafood processing sector to reduce both pathogen virulence and persistence (Sintim et al., 2010; Liu et al., 2022).

### 4.4 Future prospects

In this study, it was demonstrated that sublethal levels of BAC can lead to enhanced biofilm formation by *Vibrio* spp., along with an associated risk of inducing the VBNC state in these bacteria. This could lead to false-negative detection results when traditional culture-based methods are implemented as part of the routine quality control and food safety regimens in seafood processing facilities. Therefore, the misuse of such disinfectants could counter the desired beneficial effect of disinfectant decontamination procedures by initiating the development of pathogen populations that are more resistant and difficult to eradicate. Such bacterial adaptation will thus lead to a persistence of pathogens all along the seafood chain, from aquaculture to processing facilities, which could increase the occurrence of animal mass mortality events or food-borne disease outbreaks. Also, of great concern is that this phenomenon contributes to the rise of antimicrobial resistance with co-selection of antibiotic resistance genes and associated cross-resistance, and will thus result in increasing difficulties in therapeutic treatments (Karatzas et al., 2007; Kovacevic et al., 2013; Tandukar et al., 2013). As such, in the future, c-di-GMP blockers could be investigated in conjunction with sublethal concentrations of BAC, in order to evaluate the potential of antagonist molecules as antimicrobial agents in food industries. For instance, after a high-throughput screening, Sambanthamoorthy et al. (2012) identified several antagonists that were able to inhibit diguanylate cyclase (DGC) enzymes, that synthesize c-di-GMP, and thus reduce c-di-GMP concentration and biofilm formation of *V. cholerae*.

Moreover, in this study, we focused on mono-species biofilm, while in aquaculture and seafood industries most bacteria, including *Vibrio*, survive in polymicrobial biofilms comprised of two or more microbial residents (Chen et al., 2019; Li et al., 2021). Multispecies biofilm can promote increased tolerance against BAC, as demonstrated for combined biofilms of *L. monocytogenes* and *P.s putida* (Ibusquiza et al., 2012; Giaouris et al., 2013). Similarly, dual-species biofilms of *V. parahaemolyticus* and *Shewanella putrefaciens* were more tolerant to eradication through photodynamic inactivation compared to the mono-species biofilms (Tan et al., 2021). However, to our knowledge no study has investigated the tolerance of *Vibrio* multispecies biofilm exposed to BAC. Such knowledge will be necessary to elucidate bacterial tolerance and community dynamics in seafood industries after disinfection procedures.

## 5 Conclusion

Misuse and overuse of BAC in seafood industries may lead to the discharge of persistent BAC residues into the surrounding environments. In this study, we investigated the survival and phenotypic responses of pathogenic *Vibrio* species when exposed to these residual concentrations of BAC. Results revealed a phenotypic adaptation in different *Vibrio* bacteria, characterized by increased c-di-GMP production, leading to the development of thick biofilms. This effect was found to be strain-dependent rather than species-specific, and appeared to be influenced by inherent growth capacity in a biofilm form. This suggests that bacteria within thinner biofilms respond to BAC residues by a tolerance/resistance mechanism involving increased biomass production. Conversely, bacteria already embedded within thicker biofilms might not require adaptation to environmental stressors. Furthermore, exposure to BAC residues might amplify the presence of injured and alive cells within the biofilm. While we cannot conclude the nature of the “injured” bacteria, it is clear that BAC might induce physiological changes in *Vibrio* bacteria, with a risk of development of the VBNC state. Such bacteria are all the more threatening as they might go undetected using standard culture-based methods that are commonly used for microbiological risk assessment in aquaculture and seafood industries. False negative results arising through the presence of VBNC bacteria can lead to recurring contamination and result in disease outbreaks. These results emphasize the need for an efficient rinsing of aquaculture tanks and seafood processing equipment to avoid BAC residues, after disinfection. Future research is needed to explore the potential development of c-di-GMP blockers that might increase disinfection efficacy and help reduce microbiological risks within aquaculture and seafood industries.

## Data availability statement

The original contributions presented in the study are included in the article/supplementary material, further inquiries can be directed to the corresponding author.

## Author contributions

JM: Conceptualization, Data curation, Formal analysis, Funding acquisition, Investigation, Methodology, Project administration, Resources, Validation, Visualization, Writing – original draft, Writing – review & editing. GM: Resources, Writing – review & editing. SL: Project administration, Resources, Writing – review & editing. GB: Formal analysis, Methodology, Writing – review & editing. TE: Writing – review & editing. AJ: Resources, Supervision, Funding acquisition, Writing – review & editing. HW: Methodology, Project administration, Resources, Writing – review & editing. TB: Conceptualization, Data curation, Funding acquisition, Investigation, Methodology, Project administration, Resources, Supervision, Validation, Visualization, Writing – review & editing.
